# The Therapeutic Potential of Human Umbilical Cord Derived Mesenchymal Stem Cells for the Treatment of Premature Ovarian Failure

**DOI:** 10.1007/s12015-022-10493-y

**Published:** 2022-12-15

**Authors:** Amna Umer, Nasar Khan, David Lawrence Greene, Umm E. Habiba, Sabiha Shamim, Asma Umer Khayam

**Affiliations:** 1 R3 Medical and Research Institute Pvt. Ltd, Jahangir Multiplex, H-13 Sector, Islamabad, 44000 Pakistan; 2 R3 Medical Research LLC, 10045 East Dynamite Boulevard Suite 260, Scottsdale, AZ 85262 USA; 3 Department of Biochemistry, Quaid e Azam University, Islamabad, 44000 Pakistan

**Keywords:** Premature Ovarian Failure, Ovarian Function, Human Umbilical Cord, Mesenchymal Stem Cells, Infertility

## Abstract

Premature ovarian failure (POF) affects 1% of women under 40, leading to infertility. The clinical symptoms of the POF include hypoestrogenism, lack of mature follicles, hypergonadotropinism, and amenorrhea. POF can be caused due to genetic defects, autoimmune illnesses, and environmental factors. The conventional treatment of POF remains a limited success rate. Therefore, an innovative treatment strategy like the regeneration of premature ovaries by using human umbilical cord mesenchymal stem cells (hUC-MSCs) can be a choice. To summarize all the theoretical frameworks for additional research and clinical trials, this review article highlights all the results, pros, and cons of the hUC-MSCs used to treat POF. So far, the data shows promising results regarding the treatment of POF using hUC-MSCs. Several properties like relatively low immunogenicity, multipotency, multiple origins, affordability, convenience in production, high efficacy, and donor/recipient friendliness make hUC-MSCs a good choice for treating basic POF. It has been reported that hUC-MSCs impact and enhance all stages of injured tissue regeneration by concurrently stimulating numerous pathways in a paracrine manner, which are involved in the control of ovarian fibrosis, angiogenesis, immune system modulation, and apoptosis. Furthermore, some studies demonstrated that stem cell treatment could lead to hormone-level restoration, follicular activation, and functional restoration of the ovaries. Therefore, all the results in hand regarding the use of hUC-MSCs for the treatment of POF encourage researchers for further clinical trials, which will overcome the ongoing challenges and make this treatment strategy applicable to the clinic in the near future.

**Graphical Abstract:**

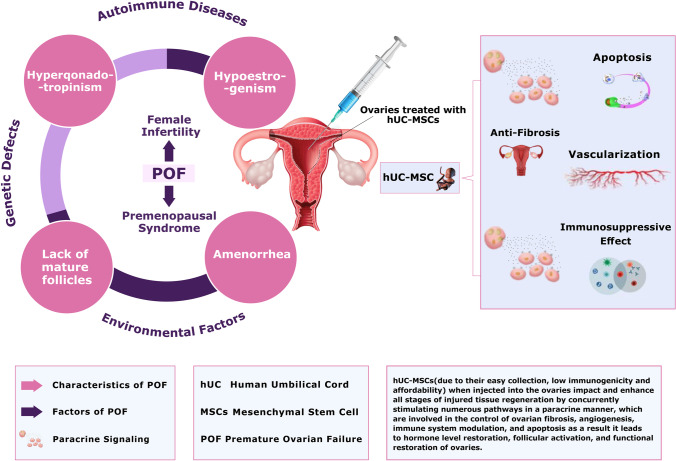

## Introduction

**Fig. 1:**
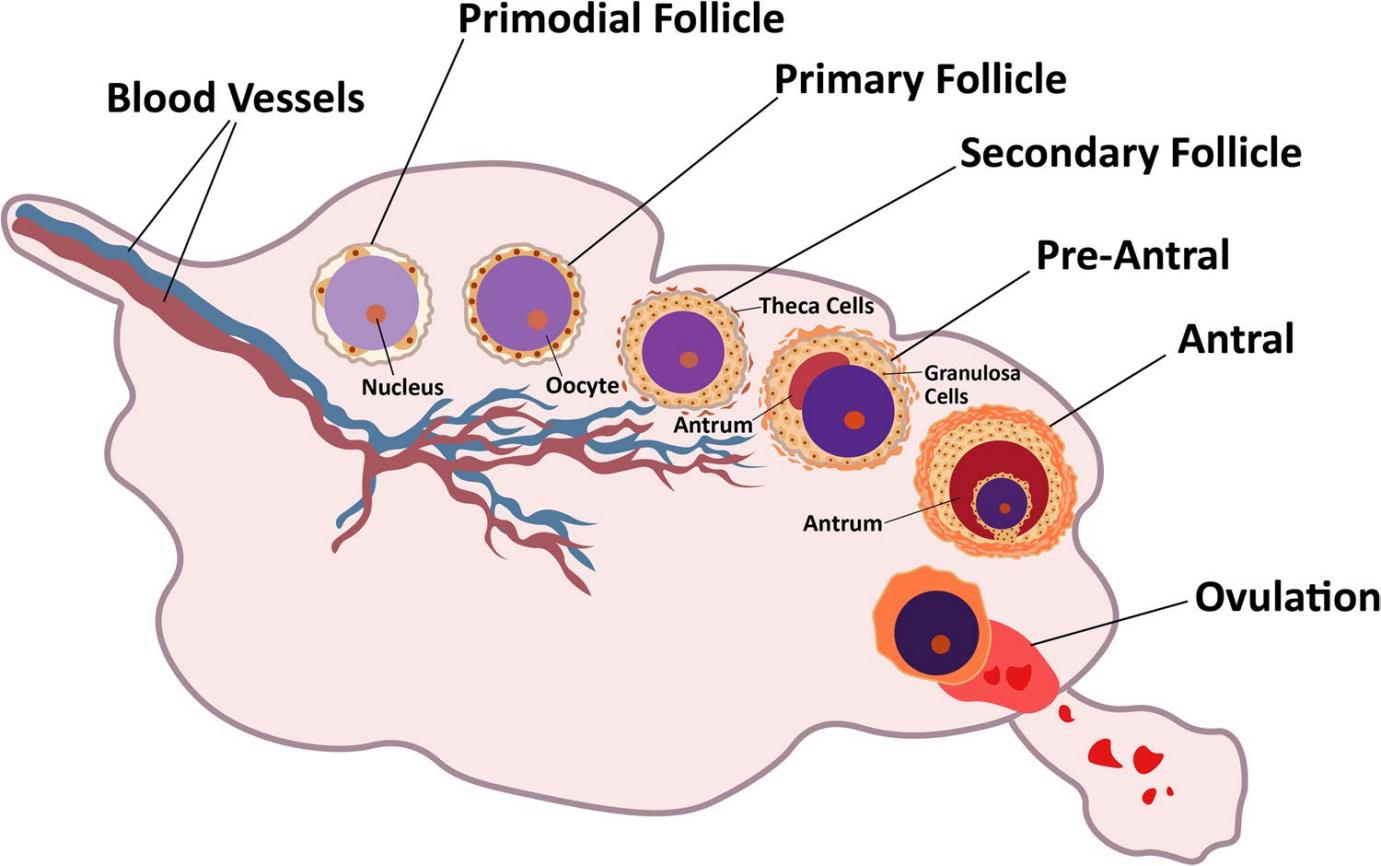
Folliculogenesis: The process involves the development of follicles (primordial) into primary, preantral, and ultimately antral follicles. Ovulation happens after antral stage

The ovaries are complex and critical reproductive organs in the female body. Multiple factors can affect the function of ovaries leading to infertility in females. The outside layer of the ovaries contains unique structures known as follicles. These follicles produce an oocyte (immature egg), which becomes mature into a fertilizable egg by a process known as folliculogenesis. Ovarian follicles include three categories of cells: oocytes, theca and granulosa. Follicle growth and development depends on the follicle-stimulating hormone (FSH) and luteinizing hormone (LH) receptors, which are found in the granulosa and theca cells. Folliculogenesis is a well-planned and regulated process. The process involves the development of primordial follicles into primary, preantral, and ultimately antral follicles. Ovulation happens after this stage (Fig. 1**)** [1]. The number of primordial follicles is restricted during a woman's reproductive life. Females are said to have entered reproductive senescence or menopause when their reserve is depleted. There are around 6 to 7 million germ cells in a female fetus. Approximately 400,000 to 500,000 primordial follicles persist by the time a girl enters adolescence. Approximately 1000 follicles each month are lost after menarche [2, 3].

Around age 50, there are only around 1000 follicles left after the number of follicles reduces to about 25,000 after age 37, and the rate of follicular loss increases. Thus, throughout a woman's reproductive life, only about 400 follicles will develop and ovulate, with the large bulk never doing so [4]. The ovaries can stop functioning due to various reproductive abnormalities that affect the ovaries causing discomfort, irregular menstrual periods, urinary problems, infertility, and pregnancy failure. Among these, Premature Ovarian Failure (POF)/Primary Ovarian Insufficiency (POI), Polycystic Ovary Syndrome (PCOS), Asherman Syndrome, Endometriosis, and Preeclampsia are the most frequent female reproductive disorders [5, 6]. Premature ovarian failure (POF), aka (POI) Primary ovarian insufficiency or early menopause, is a puzzling and complex condition. POF affects one in every 250 women under 35 years and one in every 100 women under 40 years [7, 8]. POF has significant health implications for women. The climacteric syndrome (a group of symptoms brought on by a drop in ovarian hormone levels) is the primary source of short-term consequences comparable to spontaneous menopause. POF diminishes the likelihood of a natural pregnancy significantly. Urogenital atrophy is also caused by a lack of estrogen (Lower estrogen levels in these tissues, alterations in the vagina and urethra occur). Vaginal dryness, discomfort, and itching are the most prevalent urogenital symptoms. Sexual function is further hampered by urogenital atrophy and hypoestrogenism. A decline in bone mineral density (BMD) is a hazard to POF patients. In addition to experiencing psychological anguish, investigations on POF women have found that they have a higher chance of developing neurodegenerative disorders. Generally, POF women's life span is decreased due to cardiovascular illness, osteoporosis and sexual dysfunction [9, 10]. Hypergonadotropinism and amenorrhea are also among the main POF characteristics that lead to premenopausal syndrome and female infertility [11].

## Pathophysiology of POF

**Fig. 2:**
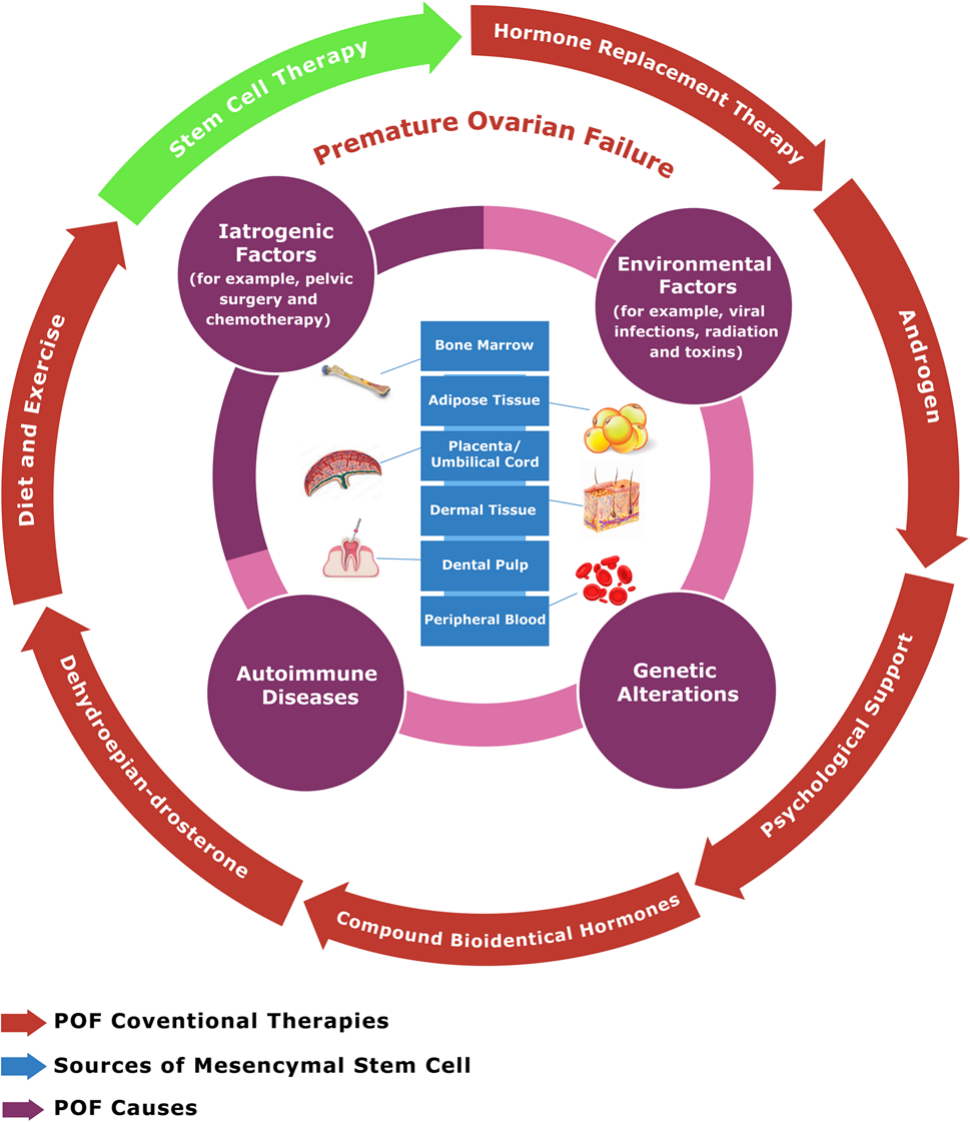
The pathogenic factors, leading causes (Iatrogenic factors, environmental stressors, genetic and autoimmune factors), and treatment options of POF (hormone replacement therapy, androgen, counseling, synthesized bioidentical hormones, Dehydroepiandrosterone, donated oocytes, exercise and diet and stem cell treatment) along with sources of mesenchymal stem cells (adipose tissue, bone marrow, dental tissues, peripheral blood, placenta/umbilical cord(UC), dermal tissue)

Two histological forms of early ovarian failure have been identified. In the first type, the ovarian follicles are entirely depleted, but in type 2, the ovary retains follicular features. According to Nelson et al. [8] the essential processes in POF are follicle depletion and follicular dysfunction. The quality of the oocytes and follicular pool can be affected by genetic, paracrine, endocrine, mitochondrial dysfunction, and metabolic variables, yet the origin of POF is unknown [12]. FSH (follicle-stimulating hormone) levels in the early follicular phase, estradiol, inhibin B, and FSH/luteinizing hormone (LH) levels are utilized to make the diagnosis [13]. A high FSH level is a clear indicator of ovarian failure. In addition to these, more hormones can be measured, which include stimulating thyroid hormone (TSH), prolactin (PRL) and anti-Müllerian hormone (AMH) [14]. Two types of inheritance patterns for POF exist: sporadic and familial [15]. Among the leading causes of POF are Iatrogenic factors, such as pelvic surgery and chemotherapy, environmental factors, such as viral infections, radiation, and toxins, autoimmune diseases with anti-ovarian antibodies causing ovarian damage, and genetic changes, such as point mutation, chromosome imbalances involving the X chromosome or autosomes (Fig. 2) [16]. Over 50% of POF cases are still idiopathic despite improvements in medicine, and they might manifest in random or familial forms [17]. Genetics may directly cause the disease or merely predispose a person to it. Regarding POF, certain elements may be found in around 20–25% of instances [18]. POF is inherited in 4–31% of cases, with an X-chromosome aberration playing a crucial part in the condition [15].

## Conventional Treatment of POF

Several treatments have been proposed owing to the complexities of POF. However, none of them has shown to be sufficiently successful in serving as a reliable first-line alternative. Treatments for POF include hormone replacement therapy, androgen, counseling, synthesized bioidentical hormones, Dehydroepiandrosterone, donated oocytes, exercise and diet, and stem cell treatment (Fig. 2). There are two different forms of sex-steroid replacement (SSR): physiological sex-steroid replacement (pSSR) and standard sex-steroid replacement (sSSR). While pSSR is used to control hormone levels in aberrant ovarian function, sSSR has improved symptoms in postmenopausal women [19]. It has been suggested that Dehydroepiandrosterone (DHEA) is a different hormonal therapy for enhancing ovarian function. Count of Antral follicles considerably increased after 16 weeks of treatment in a recent clinical trial studying the impact of DHEA on POF patients by an evaluation of ovarian response indicators, while AMH or FSH levels were not significantly impacted [20]. Ovarian theca cells and the zona reticularis of the adrenal cortex are the sources of DHEA. Almost half of the androgens are made from DHEA in tissues, created by conversion from cholesterol and estrogens. Therefore, it has a vital role in the synthesis of Testosterone (T), Estradiol (E2), and finally Estriol (E2) within peripheral tissues. DHEA is a crucial prohormone in ovarian follicular steroidogenesis because of this. Patients with POF prescribed DHEA may increase their odds of getting pregnant and reduce their risk of miscarriage [19].

The use of androgen supplementation, especially testosterone, has been studied to enhance general and sexual health [21]. Due to assertions that compounded bioidentical hormone (CBH) is a safer, more effective alternative treatment option with fewer side effects than other forms of therapy, its use has significantly expanded. Best and Triest, two popular CBH formulations, comprise of 80% estriol and 20% estradiol by weight and 10% estradiol, 80% estriol and 10% estrone, and by weight respectively [22]. Even though methods for preserving fertility for POF patients are still being developed, donor oocytes that are cryopreserved should be considered, IVF treatments using fresh donor eggs have historically had a high cumulative pregnancy rate (88%) after four rounds. In addition to fresh and new ovum donation, the utilization of cryopreserved donor oocytes is now possible thanks to the invention of vitrification, a fast-freeze process [23].

## Risks Associated with Conventional Treatments for POF

POF has a complex etiology, mainly with autosomal genetic abnormalities, X-chromosome abnormalities, immunological disorders, and enzyme deficiencies. In young individuals, the POF caused by chemotherapy medications is more evident [24]. Hormone replacement therapy (HRT) is the most prevalent POF treatment. The role of HRT in boosting fertility, on the other hand, is still debatable. Alternative therapies should be used to lessen the symptoms and risks associated with POF, as HRT is regarded as dangerous in women who have a history of ovarian cancer or breast cancer; it also raises the risk of blood clots, cancer, strokes, and other complications [25]. Egg donation is the last and most hopeful option for most POF women. However, donation of egg supplies is limited, and patients who receive these eggs will not be able to produce biological children of their own. As a result, specialists are on the lookout for more effective and innovative POF treatments. Scientists are turning to alternate therapies, such as stem cell therapy, to treat POF and other kinds of infertility due to adverse effects connected with HRT therapy used to treat POF and different types of infertility [26]. Mesenchymal Stem Cells transplantation appears to be a potential therapy option.

## Stem Cell Therapy for POF Treatment

Recently, SC therapy has been proposed as a potential substitute for treating several illnesses and has been classified as regenerative medicine. Stem cells can differentiate and self-renew to repair and restore damaged tissues or cells [27, 28]. Stem cells are unspecialized, undifferentiated cells found in stages of embryo, fetal, and adult life. Stem cells may be found in many parts of the human body and are classified based on their origin like skin and adipose, umbilical cord, amniotic fluid, placenta, and bone marrow [29]. Some of the main stem cell types like ESCs: embryonic stem cells; MSCs: mesenchymal stem cells; SSCs: spermatogonial stem cells; iPSCs: induced pluripotent stem cells have all been used in stem cell-based treatment for infertility [5, 30]. Stem cell treatment for the possible generation of ovules in women can be performed in POF patients. Several kinds of stem cells usage for the treatment of POF has been documented in recent research [25, 29–31]. The promise of a long-term replacement for damaged oocytes has prompted many to explore stem cell (SC) treatment for infertility. These transplanted SCs may establish themselves inside the ovarian tissue and restore ovarian function, as seen in a chemotherapy-induced POF mouse model [32]. For efficient ovarian rejuvenation (stimulation of follicles in the early stages), many stem cell infusion techniques, including the administration of SCs into the ovaries, laparoscopic ovarian injection, infusion through the ovarian artery (intra-arterial catheterism), TVUS-guided ovarian injection, or direct ovarian infusion/injection are used. With the stimulation of dormant follicles, these procedures may allow for a partial reversal of the ovary's aging process. Even with standard drugs, these follicles would otherwise remain in the ovary without developing [32, 33]. For women with POF who want to get pregnant, stem cell therapy is a last-resort therapeutic option. SC therapy has shown encouraging outcomes thus far, with spontaneous pregnancies occurring in women with a poor ovarian reserve who had bone marrow Stem Cell therapy [34]. Extensive investigations on SCs capable of producing oocytes have been carried out in mice and humans. These findings give a reason for optimism in developing novel POF/POI therapies. By increasing the number of primordial follicles, decreasing granulosa cell (GC) mortality, and restoring ovary sex hormone activity, SCs from diverse sources may aid and support the restoration of ovarian function [34, 35].

## Mesenchymal Stem Cells (MSCs)

Mesenchymal stem cells are multipotent mesenchymal stromal cells extracted from stromal tissues and possess plastic adhesion, self-renewal, and multi-lineage differentiation capabilities [36]. Many experts believe that transplanting mesenchymal stem cells (MSCs) is the most effective and successful way of cell therapy. These cells impact and enhance all stages of injured tissue regeneration by concurrently stimulating numerous pathways (trophic, paracrine, immunological modulation, and differentiation) [37]. MSCs originate from a variety of locations across the human body, including the bone marrow, amniotic membrane, amniotic fluid,, dental tissues, endometrium,, menstrual blood, limb buds, peripheral blood, fetal membrane and placenta, skin and foreskin, salivary gland, Wharton's jelly, sub-amniotic umbilical cord lining membrane, and synovial fluid **(**Fig. 2**)** [33, 38, 39]. MSCs provide therapeutic advantages via direct differentiation and paracrine actions, the latter of which is now regarded to be the most critical therapeutic mechanism. According to research, MSCs appear to have a more substantial influence on cell signaling than their natural regeneration abilities. Cell signaling has also been linked to regenerating and repairing dysfunctional cells [39]. Mesenchymal stem cells (MSCs) have been demonstrated in several human trials to restore ovarian function and provide a healthy treatment for women's infertility [35, 40–42]. MSCs extracted from various tissues and organs, including placenta, bone marrow, adipose tissue and endometrium, increased follicle development and improved development and quality of oocyte, ovulation, and fertility as therapies for POF [5, 43]. MSCs have homing capabilities, allowing them to accumulate spontaneously at the site of damage. As a result, MSCs migrate and attach to the wounded ovary, and multiply in response to numerous hormones and growth factors. According to these studies, MSCs boost ovarian function and aid in functional ovarian recovery. It is unknown whether MSCs differentiate into oocytes or assist stromal or follicular cells after migrating to the ovary to produce this impact [5, 35, 39–42].

## Human Umbilical Cord as a Source of Human Umbilical Cord Mesenchymal Stem Cells (hUC-MSCs)

**Fig. 3:**
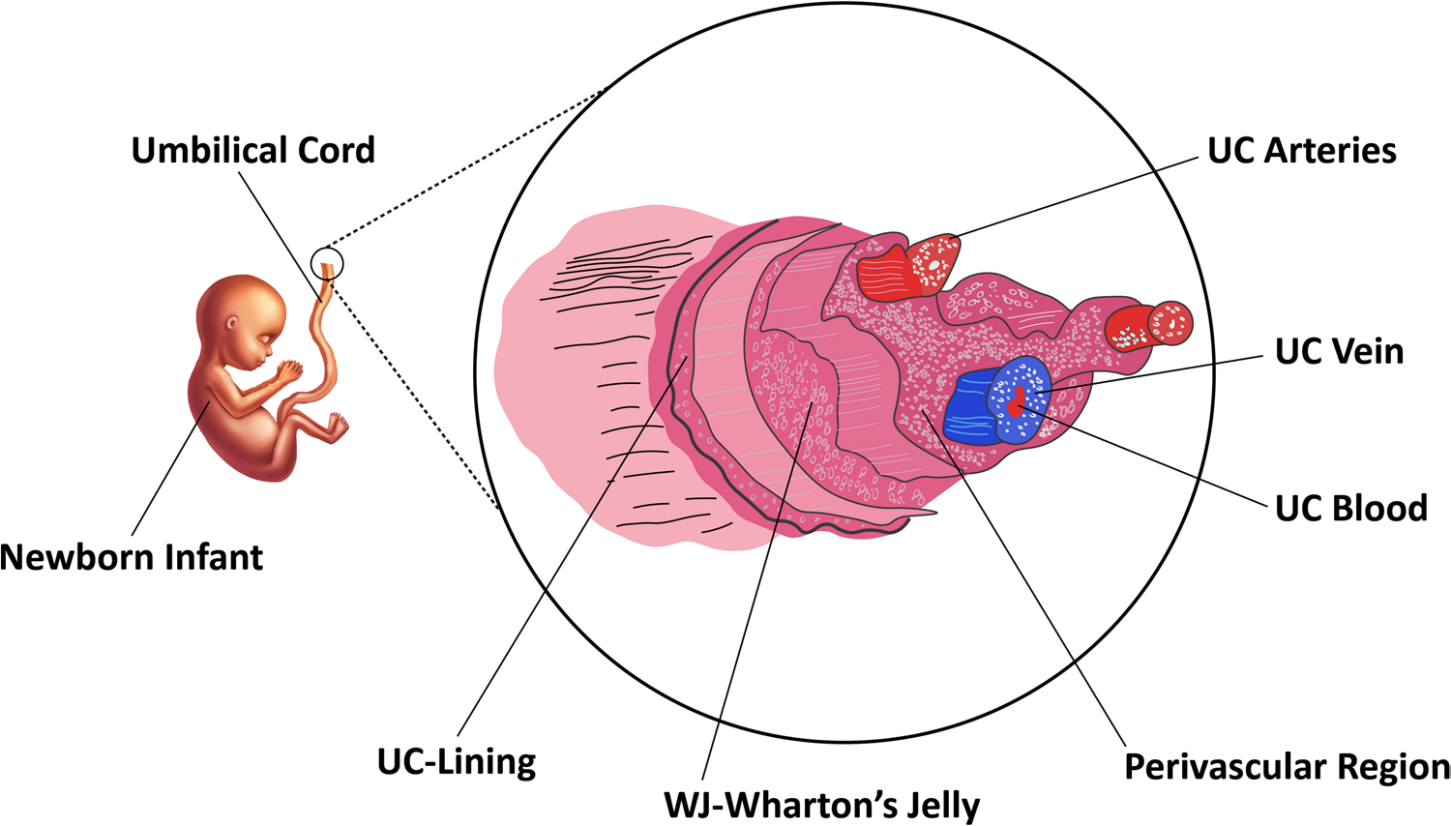
Different compartments of the umbilical cord tissue. One umbilical vein (UCV) and two umbilical arteries (UCAs) are implanted in Wharton's jelly (WJ) in the umbilical cord (UC) to form the structure of the umbilical cord (UC). The amniotic epithelium encloses mucous connective tissue. UC-Umbilical cord

The human umbilical cord is a good source of MSCs. Unlike bone marrow stem cells, MSCs from the human umbilical cord are collected more quickly and painlessly. MSCs have also been found in the vascular tissue and Wharton jelly of the umbilical cord [44]. These cells exhibit the same triple activity as bone marrow cells in tissue repair, immune response regulation, and anti-cancer capabilities. Other pluripotent or embryonic stem cells can use hUC-MSCs as a source of nutrition [45–48]. UC-MSCs can be obtained in an ethically acceptable and non-invasive manner and large numbers of UC-MSCs can be produced following expansion. Umbilical cord (UC) contains one umbilical vein (UCV) and two umbilical arteries (UCAs), both embedded within Wharton’s jelly (WJ) which is a specific mucous connective tissue covered by amniotic epithelium (Fig. 3).

hUC-MSCs can be derived from the whitish flesh of the umbilical cord, known as Wharton’s jelly (WJ-MSCs), umbilical cord blood (UC-MSCs) [49, 50] or human umbilical cord perivascular stem cells (HUCPVC) [51–53]. Even though UC blood (UCB) and the umbilical cord (UC) are commonly discarded as medical waste after delivery, they offer a rich source of stem cells. At least three types of stem cells, including endothelial progenitor cells, mesenchymal stem cells (MSCs) and hematopoietic stem cells (HSCs), have been identified from UCB. MSCs have been isolated from numerous umbilical cord compartments. MSCs have been separated from umbilical cord blood, inside Wharton's jelly and umbilical vein subendothelium: the perivascular zone, the subamnion and the intervascular site [54]. The benefits of UC–MSCs over other MSC sources are non-invasive retrieval, off-the-shelf usage, and high abundance [55].

## Advantages of hUC-MSCs

The umbilical cord is routinely discarded as medical waste after birth; therefore, it rules out ethical concerns about their access as the procedure is non-invasive. UC-MSCs, like MSCs from other sources, have a unique ability to self-renew while preserving their multipotency (the ability to develop into osteocytes, adipocytes, chondrocytes, hepatocytes, and neurons) [53]. Stem cells increase ovarian function through paracrine actions rather than developing into particular cells [56]. Different growth factors, cytokines, signaling lipids, regulatory miRNAs and messenger RNAs (mRNAs), are all found in the vesicles released by mesenchymal stem cells (MSCs). Cellular signaling, Cell-to-cell communication, and changes in tissue or cell metabolism are all affected by these variables [57]. The immunomodulatory characteristics of UC-MSCs have also grabbed people’s interest. UC-MSCs are now highlighted as a potentially adaptable regenerative medicine and immunotherapy strategy [53]. UCMSCs have few specific properties that they maintain namely, quick self-renewal, low risk of tumor, low chance of immune rejection, less ethical concerns, non-invasive and painless collection, and easy production in bulk.

## Paracrine Effects of hUC-MSCs

**Fig. 4:**
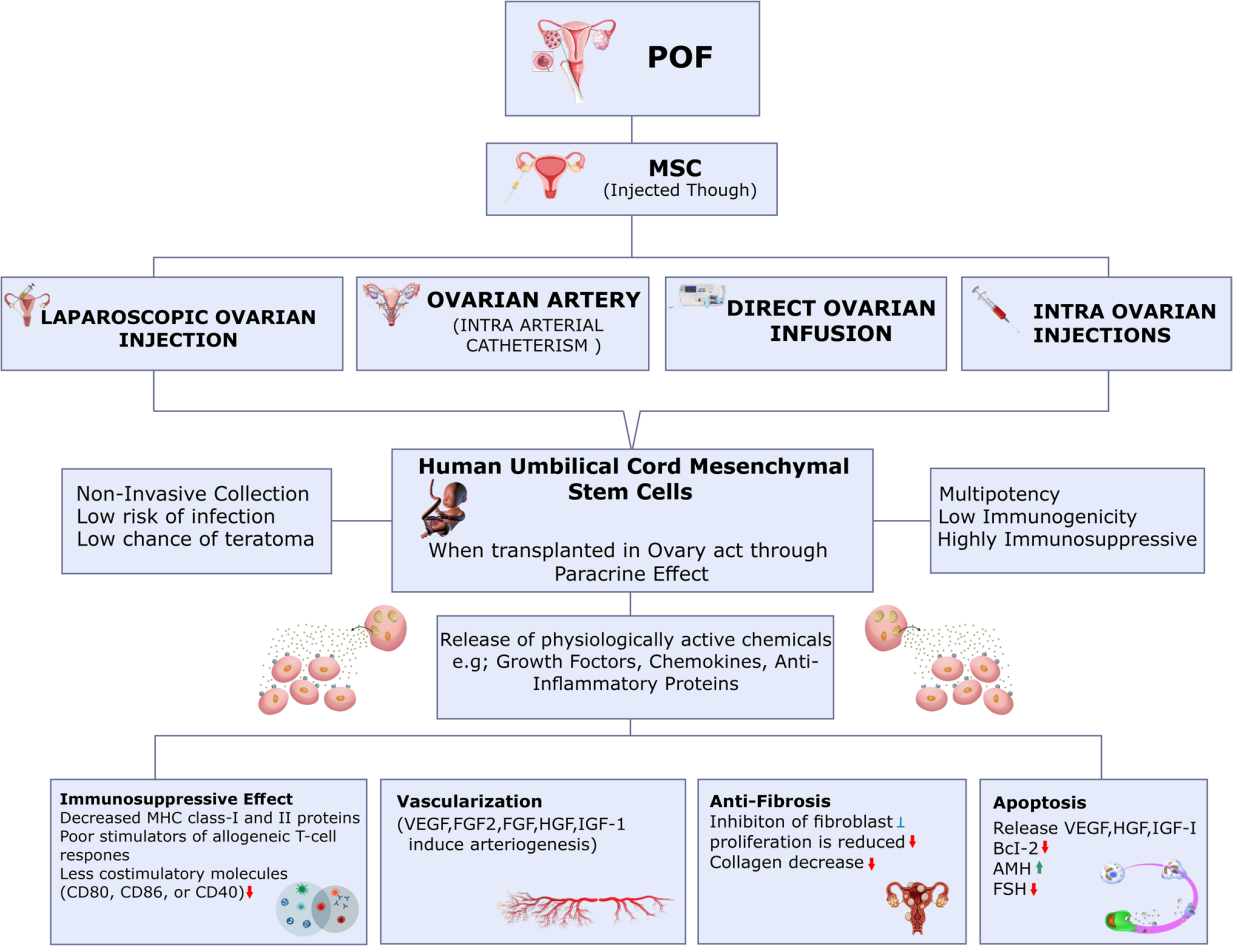
Proposed mechanisms of ovarian damage treatment using stem cells. The flow diagram represents how premature ovarian failure is treated by injecting MSCs through various methods and as a result MSCs act through paracrine signaling and are involved in the control of ovarian fibrosis, angiogenesis, immune system modulation, and apoptosis. Premature Ovarian Failure (POF), Mesenchymal Stem Cells (MSCs), Vascular Endothelial Growth Factor (VEGF), Fibroblast Growth Factor 2 (FGF2), Fibroblast Growth Factor (FGF), Hepatocyte Growth Factor (HGF), Insulin-Like Growth Factor Factor 1 (IGF-1), Anti-Müllerian hormone (AMH), Follicle-stimulating hormone (FSH).

hUC-MSCs have a high cytokine secretion profile, allowing them to proliferate faster, differentiate more effectively, and have low immunogenicity. Interleukins (IL), colony-stimulating factors (CSF), tumor necrosis factor (TNF), interferons (IFN), chemokines and TGF are among them. These cytokines are involved in homing, anti-inflammatory, migration, anti- pro-angiogenesis, and apoptosis pathways in UCMSCs. It has been demonstrated in several investigations that these UCMSCs can repair compromised ovarian function by producing cytokines and other factors involved in proliferation and tissue formation [58]. Here is a proposed explanation of the fundamental mechanism of action that stem cells are thought to have (Fig. 4). It was reported that the ovaries treated with hUC-MSCs showed that vascularization in the ovarian niche was increased in the cyclic healing process and may be helpful for ovarian recovery in the context of angiogenesis. In vivo and in vitro, factors like (HGF) hepatocyte growth factor, (VEGF) Vascular Endothelial Growth Factor, insulin-like growth factor-1(IGF-1) and (FGF-2) Fibroblast Growth Factor-2 generated by these cells have been reported to induce arteriogenesis [59]. When VEGF and HGF are combined, vascular diameter is enhanced. HGF helps with vascular area expansion, whereas VEGF enhances the area, length, and several branch points of the induced vessels [60].

## Immunosuppressive Effects of hUC-MSCs

hUC-MSCs are an abundant source of MSCs that display stem cell-specific markers and can be differentiated into various types of mesodermal cell for immune response modulation and tissue repair. The poor immunogenic qualities are related to decreased production of MHC class I and class II proteins, which are required for adaptive immunity. MSCs interact with various immune cells; in vitro, they are poor stimulators of allogeneic T-cell responses [61]. The T-cell receptor recognizes MHC molecules in combination with an antigen that is on the surface of an (APC) antigen-presenting cell in the first signal [62, 63]. Following that, T-cell activation requires the CD28 on the T cell with CD86 or CD80 (B7 superfamily) interaction on the APC through a co-stimulatory signal. MSCs have low quantities of MHC-I molecules on their surface, but none of the MHC-II or co-stimulatory molecules like CD80, CD86, or CD40. Because MSC lack MHC class II, they can avoid being recognized by alloreactive CD4 + T cells. It is also worth noting that co-stimulatory chemicals are not present. Any remaining T cell receptor contact on Th cells will result in anergy and tolerance rather than an allogeneic response [64, 65]. MSCs have been shown to exhibit immunoregulatory capabilities in the ovarian niche by controlling the populations of macrophages, cytokines and regulatory T lymphocytes. MSCs interact with innate and adaptive immune systems immune cells to display immunoregulatory properties [60]. MSCs control T-cell growth and function [66]. MSCs only carry out immunoregulatory functions in the restricted target tissue; they have no systemic impact. Therefore, they do not inhibit the immune system systemically.

Furthermore, the hUC-MSCs were reported to restore ovarian function in POF mice via modulating the ratio of (Th1/Th2) T-helper-1 and T-helper-2 cytokines and the number of (NK) natural killer cells [67]. Moreover, the ovarian function is also improved through regulating cytokines like TGF-β, Treg cells, and interferon-g (IFN-g) [68]. By releasing hormones, cytokines, inhibiting apoptosis-related genes and upregulating proliferation-related genes, MSCs can decrease GC death and enhance GC proliferation [69]. Wang et al. [70] showed that the transplantation of MSCs might help in the restoration of ovarian function by upregulating and FSHR and AMH expression in POF mice's GCs, inhibiting GC apoptosis and follicular atresia. Another study by Fu et al. [25] showed that by releasing VEGF, IGF-1, HGF and upregulating Bcl-2 expression, BMSC transplantation might minimize GC apoptosis and enhance ovarian function. Ovarian cell proliferation and apoptosis rates are linked to ovarian function hence controlling MSC efficacy in POF therapy. A drop in collagen levels following the transplantation of MSCs has also been seen in connection to the reduction of fibrosis, indicating that the mechanism of action of these cells in the healing of ovarian injury may also entail an antifibrotic impact [71]. In fact, fibroblast proliferation may be inhibited and extracellular matrix deposition levels may be reduced by MSCs [72]. Also, MSCs have an immune-regulating impact, making them a prospective therapy option for immune-mediated POF. As a result, more comprehensive processes are required to boost therapeutic efficacy of MSCs and provide a foundation for their clinical application [25].

## Pre-Clinical Trials for the Treatment of POF using hUC-MSCs

Although there are few studies on the usage of UCMSC in POF therapy, it has been shown that UCMSC transplantation may be able to restore ovarian function via the paracrine pathway. According to the findings, UCMSC expressed more proteins engaged in the epigenetic pathway, transcription pathway, DNA modification, cell signaling, and protein modification pathway. UCMSCs can swiftly heal the ovarian injury by stimulating the development of granulosa cells through these pathways. Some ovarian stem cell function proteins, such as Ly6a (Sca-1) and AKR1C18 [73–75], can be increased by UCMSCs. UCMSCs can release powerful combinations of trophic substances that alter the environment's molecular composition and elicit reactions from resident cells. Mesenchymal Stem Cells (MSCs) have been reported for their ability to heal damaged ovaries caused by chemotherapy [76]. UCMSCs can elude immunological reactivity even in allogeneic transplants because of lower expression of human leukocyte antigen (MHC-I) major histocompatibility complex I and lack of MHC-II molecules indicating that transplanting UCMSCs causes minimal to no immunological rejection [77].

Research also demonstrated that stem cells can prevent apoptosis by secreting stanniocalcin-1 and other paracrine substances [78–80]. Some pre-clinical studies indicate that hUC-MSCs were reported to restore ovarian function in POF mice via modulating the ratio of (Th1/Th2) T-helper-1 and T-helper-2 cytokines and the number of natural killer NK cells [67] and through regulating TGF-β, Treg cells, and (IFN-g) interferon-g cytokines [68]. Angiogenic growth factors that MSCs may release include TGF-, hepatocyte growth factor (HGF), (VEGF) vascular endothelial growth factor, and placental growth factor, and the protective effect of transplanted UCMSCs is mediated by factors that play a role in the restoration ovarian function by the secretion of NGF and TrkA to inhibit GC apoptosis (PGF) [81]. MSCs can increase GC proliferation and decrease GC death by producing cytokines and hormones, upregulating proliferation, and inhibiting apoptosis-related genes [69]. MSC transplantation restored ovarian function by upregulating AMH and FSHR expression in POF mice's GCs, inhibiting GC apoptosis and follicular atresia by releasing VEGF, HGF, and IGF-1 upregulating Bcl-2 expression [82].

A recent study on the POF rat model demonstrated that the indicators underlying ovarian function were restored by UCMSC transplantation through the TGF-β1/Smad3, Hippo, AMPK/mTOR and PI3K/Ak signaling pathways; UCMSCs could influence the development of ovaries, growth of follicles, cell proliferation and differentiation of granulosa [71, 83]. The PI3K pathway was shown to be activated by human UCMSCs when they were used for the restoration of ovarian function. This lowered monosaccharide content and enhanced lipid metabolism. After further investigation on a complete signaling pathway analysis of the molecular processes of repairment and damage on the injection of UCMSCs to rats with POF models, this work may provide a foundation for the therapeutic application of UCMSC to treat POF [84]. Another report on ovarian function restoration demonstrated that hUC-MSC transplantation could have a positive and preventative effect on rats using the POF model. (Deng et al., 2021)It was also seen that UC-MSCs significantly enhanced the function of ovaries in chemotherapy-induced POF by inhibiting apoptosis by reducing inflammation via activation of AKT and P38 pathways. These hUC-MSCs were injected into POF rats' ovaries, reducing sexual cycle dysfunction, enhancing serum hormone expression, and restoring ovarian function [85] (Deng et al., 2021). Overall ovarian cell proliferation and apoptosis rates are linked to ovarian function thereby controlling MSC efficacy in POF therapy. Also, MSCs have an immune-regulating impact, making them a prospective therapy option for immune-mediated POF. However an evaluation of more comprehensive processes are needed to increase the therapeutic effectiveness of MSCs and provide a foundation for their clinical application field.

## Clinical Trials Regarding the Treatment of POF using hUC-MSCs

Numerous clinical research has been conducted to address the issues primarily related to pregnancy since POF has significant health implications for women. The primary outcomes of the disease have also been analyzed, such as the size of the ovaries; Serum hormone levels such as Anti-Müllerian hormone (AMH), luteinizing hormone (LH), follicle-stimulating hormone (FSH), and estradiol (E2). Secondary outcomes were also studied in clinical studies, such as the number of antral follicle counts (AFC) and dominant follicles (DFC) every month; the number of babies born and pregnancies; the quantity of harvested oocytes, matured oocytes, high-quality embryos, the percentages of clinical pregnancy, miscarriages, or live birth for ICSI /IVF cycles; as well as the frequency of unfavorable incidents such temperature, rash, vaginal bleeding, headaches, infectious diseases, abnormal liver functions, neoplasms, and aberrant renal function were also studied [86]. The first clinical studies were conducted on people using MSCs from bone marrow (BM) using SCs, through iliac crest aspiration for cell collection, Stem cell isolation, and invitro culture methods [72]. A stem cell therapy resulting in the live birth of a 2.7 kg female baby by a 45-year-old perimenopausal lady was reported by Gupta et al. [34]. Positive results with a similar technique, with 10 POF younger women, showed a restoration of menstruation in two patients and a pregnancy with live birth [87]. Similar to another study in 30 POF women (18–40 years old) resulted in one pregnancy and improved symptoms in fields [88].

According to research, BMDSC boosts ovarian vascularization, stromal cell proliferation, and cell apoptosis to enhance human and mouse follicular growth [89]. Based on this knowledge, a team created future pilot research on 17 POF women to examine the effects of autologous stem cell ovarian transplant (ASCOT) on ovarian reserve. As a result, six pregnancies and three different newborns were accomplished, and 81.3 percent of women had improved ovarian function biomarkers (AMH and AFC) [90]. According to the findings by Liu et al. [91], ASCOT may have enhanced the proliferation of preexisting follicles by mediating the presence of certain factors released by stem cells, namely FGF-2 and THSP-1, during aphaeresis [92]. It is crucial to note that numerous Randomized Controlled Trials (RCT) involving POF women are testing multiple additional stem cell origins across the globe. There are now eleven clinical studies using MSCs as a treatment for POI/POF patients globally, according to data from ClinicalTrials.gov. Out of these eleven studies, five include transplanting Human Umbilical Cord MSCs. Clinical trials on the transplantation of UCMSCs into POI/POF patients are still ongoing, and more research is still needed to determine if UCMSCs are effective in treating POI/POF. A Clinical Study titled “Human Umbilical Cord Mesenchymal Stem Cells in the Treatment of Premature Ovarian Insufficiency case studies” was submitted by Li-jun Ding, Nanjing University, in April 2022 (NCT05308342) (https://www.clinicaltrials.gov/). This was an interventional, single-center, randomized, controlled prospective study. To determine the efficiency and safety of hUC-MSC in the treatment of patients (Married Females, 20 years old ≤ age < 40 years old, having an average diameter of each ovary > 10 mm; with premature ovarian failure were enrolled and treated with hormone replacement combined with transplantation of hUC-MSC into ovaries. UC-MSCs were injected into the ovary of patients under transvaginal ultrasonographic (TVUS)-guidance by medical physicians. This ongoing study provides promising templates for future similar studies. However, most of them are still in progress. Thus, no outcomes have been announced (Table 1).

**Table 1:** Data from pre-clinical and clinical research supporting the efficacy of using hUC-MSCs to treat POF

#	Regenerative Factor	Delivery method	Year	Study sample	Dosage	Title of study	Research Outcome	Reference
1	UC-MSCs	Intra-ovaries injections	2013	Mice	2 × 105	“The Therapeutic Potential of Umbilical Cord Mesenchymal Stem Cells in Mice Premature Ovarian Failure”	Recovery in Sex Hormone (E2) Levels as a result improve Ovary Function. Granulosa Cells Apoptosis is reduced by UCMSCs	(S. Wang et al. 2013)
2	hUC-MSCs	Intravenous	2016	Rat	5 × 106	“Human Umbilical Cord Mesenchymal Stem Cells Therapy in Cyclophosphamide-Induced Premature Ovarian Failure Rat Model”	Recovered disturbed hormone secretion and folliculogenesis and reduced ovarian cell apoptosis	(Li et al. 2016)
3	hUC-MSCs	Intravenous	2019	Mice	1 × 106	“Distribution of the CM-Dil-Labeled Human Umbilical Cord Vein Mesenchymal Stem Cells Migrated to the Cyclophosphamide-Injured Ovaries in C57BL/6 Mice”	Increased chemokines in the damaged and inflamed tissues of ovaries affect the growth of blood vessels and prevent apoptosis	(Jalalie et al. 2019)
4	hUC-MSCs	Intra-ovaries injections	2019	Mice	2 × 105	“Transplantation of umbilical cord-derived mesenchymal stem cells on a collagen scaffold improves ovarian function in a premature ovarian failure model of mice."	Increased CD31 expression (promote ovarian angiogenesis)	(Yang et al. [81])
5	hUC-MSCs	Intravenous	2019	Rat	5 × 106	“Umbilical Cord Mesenchymal Stem Cell Transplantation Prevents Chemotherapy-Induced Ovarian Failure "	UCMSC Transplantation Improved Pregnant Rate and Embryos Numbers of POF Rats	(Zheng et al. 2019)
6	hUC-MSCs	Tail vein injection	2020	Rats	0.25 × 106 1.00 × 106 4.00 × 106	“Mesenchymal Stem Cell Therapy Using Human Umbilical Cord in a Rat Model of Autoimmune-Induced Premature Ovarian Failure"	Cellular proliferation of the ovary is promoted, cell apoptosis of ovary is reduced, the expression of related genes is regulated	(Z. Wang et al. [69])
7	hUC-MSCs	Intravenous/Intra-ovarian injection	2022	Rats	2 × 106	“Umbilical Cord Mesenchymal Stem Cells Ameliorate Premature Ovarian Insufficiency in Rats”	The decrease in number of apoptotic granulosa cells The increase in number of follicles, and the serum level of E2 and AMH was increased, promoting the ovarian function restoration	(Zhang et al. [84])
8	hUC-MSCs	Unknown	2012	Human n = 40	Unknown	“Stem cell therapy combined hormone replacement therapy in patients with premature ovarian failure."	Not available	NCT01742533
9	hUC-MSCs	Bilateral ovarian injection	2015	Human n = 23	1 × 107	”Transplantation of hUC-MSCs with injectable collagen scaffold for POF”	Not available	NCT02644447
10	hUC-MSCs	Intraovarian injection under ultrasonic	2016	Human n = 320	unknown	“hUC-MSCs transplantation in women with POF”	Not available	NCT03033277
11	hUC-MSCs	Intravenous injection	2018	Human n = 12	3 × 107 6 × 107 9 × 107	“The safety and efficiency study of MSC in premature ovarian insufficiency”	Not available	NCT03816852
12	hUC-MSCs	Intraovarian injections	2020	Human n = 61	1 × 107	“Clinical analysis of human umbilical cord mesenchymal stem cell allotransplantation in patients with premature ovarian insufficiency”	(VEGF), (FGF-2), (EGF), (IGF-1), (HGF), secreted by MSCs, upregulates (Bcl-2), reducing apoptosis in granulosa cells, facilitating neovascularization, and inhibiting ovarian aging	(Yan et al. 2020)
13	hUC-MSCs	Intraovarian injection under ultrasonic	2022	Human n = 66	10 × 106	“Clinical Study of Human Umbilical Cord Mesenchymal Stem Cells in the Treatment of Premature Ovarian Insufficiency”	Not available	NCT05308342

Premature Ovarian Failure (POF), Human Umbilical Cord Mesenchymal Stem Cells (hUC-MSCs), Mesenchymal Stem Cells (MSCs), Vascular Endothelial Growth Factor (VEGF), Fibroblast Growth Factor 2 (FGF2), Fibroblast Growth Factor (FGF), Hepatocyte Growth Factor (HGF), Insulin-Like Growth Factor Factor 1 (IGF-1), Estradiol (E2), Anti-Müllerian hormone (AMH), Follicle-stimulating hormone (FSH)

A study suggested that UCMSC's effects on reproduction may be caused by an increase in ovarian angiogenesis, which encourages granulosa cell proliferation and the creation and development of follicles. Four patients became pregnant following in vitro fertilization and embryo transfer using the UCMSC therapy (intraovarian injections of UCMSCs once to three times), and their offspring developed usually. This treatment successfully restored ovarian function in 61 women without causing severe adverse events [86].

Interestingly, research indicates that MSCs show promise as a treatment for various illnesses. Clinical and pre-clinical research is being done on the impact of MSCs on POF. According to the preliminary study findings, systemic administration of MSCs in clinical settings has significant therapeutic potential and will offer hope to POF patients. The clinical use of MSCs in POF will be available soon as a result of several successful clinical trials that have been accomplished [65]. hUC-MSCs can move into inflamed or injured tissue and develop into three separate germ layers, aiding in tissue healing [25]. Through various methods, MSCs obtained from multiple sources exhibit the same therapeutic effects in the therapy of POF. Even in elderly POF patients, MSCs offer to promise clinical transformation and practical potential for restoring reproductive function in POF patients. The molecular mechanism of POF is currently poorly understood, and this is a significant research problem for thoroughly assessing the efficacy and safety of transplantation of MSC, particularly the longer-term effect on parents and offspring [31, 93]. Based on previous investigations and core mechanistic studies, clinical study studies could significantly improve the treatment of ovarian dysfunction. There are more than 300 clinical trials for MSC therapy. Multipotent mesenchymal stromal cells (MSCs) are being investigated for nearly all therapeutic applications imaginable, according to more than 1050 clinical studies listed at FDA.gov. Numerous companies have commercialized or are currently commercializing MSC-based treatments. However, most MSC therapies have fallen short of meeting their primary efficacy end goals in clinical stages.

## Concluding Remarks

Many couples experience grief and despair due to premature ovarian failure that contributes to infertility. Due to the complexity of this illness, no single therapy has been suggested; instead, combination treatments with fewer complications have been used. Though not particularly efficient, hormone therapy is typically used by POF patients to manage the symptoms. Over the past ten years, many stem cell types with therapeutic promise have been employed to treat POF. Most studies demonstrated the effectiveness of stem cells in treating POF, with evidence showing that these cells may develop into ovarian follicles and regain ovarian function. BMDSC received the most excellent attention globally for treating various diseases, including POF. However, severe drawbacks in clinical trials, including discomfort associated with their acquisition, aging-related declines in transplant efficacy, and the potential for immunological rejection alternatives to BMSCs, are suggested, such as Mesenchymal stem cells [85]. According to the preliminary findings of pre-clinical, clinical, and laboratory-based investigations, MSC is promising for treating POF. Numerous studies suggest that human mesenchymal stem cells offer promising clinical transformation and application potential in POF patients to fundamentally restore ovarian function because they are easily accessible and differentiate into most tissues. MSCs obtained from multiple sources and methods exhibit the same therapeutic effects in restoring reproductive function in POF patients [93, 94]. The therapeutic action of MSCs is generally regulated by a complex web of biological processes rather than a single component. Following MSC migration to the damaged ovary, paracrine effects control ovarian cell proliferation, induction of apoptosis and autophagy, immunization, fibrosis, and oxidative stress, low immunogenicity, variety of origins, and affordability takes place.

The mesenchymal stem cells that are extracted from the human umbilical cord (hUC-MSCs) comprise umbilical cord tissue-derived stem cells, and they retain not only mesenchymal stem cells' essential characteristics but also have a strong capacity for proliferation and differentiation. UCMSCs are exceeding becoming an acceptable substitute for BMSCs in therapeutic settings due to their several benefits, such as a Non-invasive collecting approach for allogeneic or autologous usage and their safe mode of harvesting that reduces the chances of an ethical conflict. The reason is hUC-MSCs pose no danger or impairment to the mother or the child, and 90% percent of human cords can quickly be separated to provide their considerable amounts. They are a dependable source of MSC for therapeutic use, given that they can be frozen and thawed, clonally propagated, altered to generate foreign proteins, and cultured in culture. Their Multipotent characteristics, with a Low chance of teratoma (as they are not tumorigenic and reside in the mesenchymal tissues) and a lower immunological competency with high immunosuppressive capacity than BMSCs, seems to favors them great potential as a prospective therapy option for POF.

By enhancing hUC-MSCs' capacity to survive, and an effective delivery method to the ovary, new techniques are being developed to boost their effectiveness and render them more promising for use in clinical settings. Overall, the recent findings demonstrated in pre-clinical study by Zhang that on UCMSC transplantation, the indicators underlying ovarian function had been restored, depicting an improvement in the ovarian function of rats [95]. This study might offer a base for the clinical use of UCMSC to treat POF. However, a thorough signaling pathway investigation of the molecular mechanisms of damage and repair when UCMSCs are administered in POF-modeled rats is required. The clinical use of UCMSCs in POF needs further studies in a clinical setting to elucidate their mechanism of therapeutic action, and using UCMSCs without proper supervision could have negative repercussions. A critical scientific challenge remains in fully and thoroughly assessing the efficacy and safety of UCMSC transplantation, particularly the long-term effects on mother and child.

## Challenges and Future Prospects

As apparent as it may seem that UCMSC-based therapy seems to be gaining momentum, and we expect many new pre-clinical and clinical investigations will likely be conducted when additional stem cell characteristics are discovered, it still faces some challenges. Considering the clinical application of UCMSCs has a prerequisite of safety and efficacy for human cell-based therapy, several concerns still need to be addressed as current studies lack in-depth studies and need more future work. Comprehensive experimental and mechanistic studies are to be carried out in pre-clinical and clinical settings to address many technical issues. These issues include the number of cells to be transplanted, the window of time chosen for transplantation, the length of the therapeutic effect, the rate of injection, the frequency of transplantation, and the administration technique. However, all the participants in recent clinical studies received intra-ovarian injections of UCMSCs and displayed zero severe side effects/complications related to the treatment. In the studies, the intravenous injection might be a method of choice with far less invasiveness and a relatively short recovery time. Future randomized clinical studies should include an appropriate control group for a realistic assessment of the technique's effectiveness in a chosen group of patients. Since few in-depth investigations are available, deciphering POF's molecular mechanism remains a significant research challenge. Using adequate mechanistic investigations, we can then accurately assess the effectiveness and safety of UCMSC transplantation, particularly its long-term effects on parents and children.

Although UCMSCs only carry out immunoregulatory functions in the target localized tissue and do not lead to systemic immune function suppression, the ability of MSC-based therapies to impair immune function and promote tumor growth is another risk concern. Nevertheless, the lack of adverse effects and the safety of MSCs-related therapies is a central priority of fundamental research and clinical trials. Whether large dosages of UCMSCs produce malignant transformation must be determined to rule out tumor initiation and progression caused by these cells.

While there have been no cases of MSC-related malignancies developing in human patients, it is still possible for tumors to occur because of their propensity to encourage metastatic growth. Areas of tissue damage and inflammation draw MSCs, and as part of their regular healing activity, MSCs can home into the cancerous microenvironment. Despite their paracrine functions and the production of numerous trophic factors, MSCs, which have immunosuppressive qualities in the tumor microenvironment, can be regulated by tumor cells and, in turn, control the development, proliferation, and metastasis of tumor cells. However, studies show that Wharton's jelly stem cell (hWJSC) lysates were proven to reduce tumor development when MSCs were used as a therapy, displaying tumor-suppressive effects via enhancing pro-apoptotic Bax and lowering/downregulating anti-apoptotic Bcl-2 and SURVIVIN genes on breast adenocarcinoma, osteosarcoma, and ovarian carcinoma cells [96].

The absence of standard systematic protocols for MSC isolation, culture, identification, preparation, and administration methods leads to inconsistent outcomes in therapeutic settings. The International Society put out a set of core standards for using human MSCs for Cellular Therapy. Even though these criteria are not entirely implemented in clinical trials, they serve as a crucial starting point for creating more specific clinical application standards. Developing standards for MSC preparation in clinical practice is necessary since the underlying therapeutic processes vary depending on the cell source, the particular illness, and the intended usage. Researchers suggest efficient resource management promotes mechanistic research on MSCs and direct clinical trials to produce high effectiveness in the future [97]. The potential of Induced pluripotent stem cells (iPSCs)-based approach to overcome the primary constraints of current, donor-derived MSC production processes seems promising, as it makes it possible to create an almost infinite number of MSCs from a single blood donation [98].

The encouraging outcomes of several productive laboratory-based studies and the pre-clinical or clinical results of MSCs-mediated treatment have prompted researchers to examine MSCs-based therapeutic strategies thoroughly. After standardizing the preparation and verifying the safety of application in clinical investigations, MSCs-derived treatments will eventually be used to address POF or numerous difficult clinical conditions.

## Data Availability

All the data and material of this manuscript will be accessible to the readers.
